# Living with low muscle mass and its impact throughout curative treatment for lung cancer: A qualitative study

**DOI:** 10.1371/journal.pone.0304003

**Published:** 2024-07-29

**Authors:** Nicole Kiss, Anna Ugalde, Carla M. Prado, Linda Denehy, Robin M. Daly, Shankar Siva, David Ball, Steve F. Fraser, Lara Edbrooke

**Affiliations:** 1 Institute for Physical Activity and Nutrition, Deakin University, Geelong, Victoria, Australia; 2 Department of Health Services Research, Peter MacCallum Cancer Centre, Parkville, Victoria, Australia; 3 Institute for Health Transformation, Deakin University, Geelong, Victoria, Australia; 4 Department of Agricultural, Food and Nutritional Science, University of Alberta, Edmonton, Alberta, Canada; 5 Physiotherapy Department, University of Melbourne, Parkville, Victoria, Australia; 6 Lung Service, Peter MacCallum Cancer Centre, Parkville, Victoria, Australia; 7 Sir Peter MacCallum Department of Oncology, University of Melbourne, Parkville, Victoria, Australia; Wits University: University of the Witwatersrand Johannesburg, SOUTH AFRICA

## Abstract

**Objectives:**

To 1) explore the experience of patients with lung cancer with low muscle mass or muscle loss during treatment and the ability to cope with treatment, complete self-care, and 2) their receptiveness and preferences for nutrition and exercise interventions to halt or treat low muscle mass/muscle loss.

**Methods:**

This was a qualitative study using individual semi-structured interviews conducted using purposive sampling in adults with a diagnosis of non-small cell lung cancer (NSCLC) or small-cell lung cancer (SCLC), treated with curative intent chemo-radiotherapy or radiotherapy. Patients who presented with computed tomography-assessed low muscle mass at treatment commencement or experienced loss of muscle mass throughout treatment were included. Data were analysed using thematic analysis.

**Results:**

Eighteen adults (mean age 73 ± SD years, 61% male) with NSCLC (76%) treated with chemo-radiotherapy (76%) were included. Three themes were identified: 1) the effect of cancer and its treatment; 2) engaging in self-management; and 3) impact and influence of extrinsic factors. Although experiences varied, substantial impact on day-to-day functioning, eating, and ability to be physically active was reported. Patients were aware of the overall importance of nutrition and exercise and engaged in self-initiated or health professional supported self-management strategies. Early provision of nutrition and exercise advice, guidance from health professionals, and support from family and friends were valued, albeit with a need for consideration of individual circumstances.

**Conclusion:**

Adults with NSCLC with or experiencing muscle loss described a diverse range of experiences regarding treatment. The types of support required were highly individual, highlighting the crucial role of personalised assessment of needs and subsequent intervention.

## Introduction

Lung cancer was the leading cause of cancer-related mortality globally, responsible for 18% of all cancer deaths, and the second most commonly diagnosed cancer worldwide in 2020 [1]). Curative intent radiotherapy, with or without chemotherapy, is a common treatment course for advanced stage non-small cell lung cancer (NSCLC) and limited stage small cell lung cancer (SCLC) [2]). Chemo-radiotherapy can lead to significant acute toxicities, including oesophagitis and nausea, contributing to nutritional and functional deterioration secondary to muscle loss, with a subsequent negative impact on health-related quality of life and survival [3–5]).

Low muscle mass affects up to 47% of people with NSCLC prior to commencing chemotherapy and up to 61% prior to chemo-radiotherapy [6, 7]). In addition to pre-existing low muscle mass, loss of muscle mass may also occur throughout treatment. A systematic review and meta-analysis of observational studies in people with advanced NSCLC treated with chemotherapy found a mean reduction in skeletal muscle index of 0.45 kg between pre- and post-chemotherapy [8]). Few studies have investigated the extent of muscle loss in people with lung cancer treated with radiotherapy, but one study including 41 people with NSCLC reporting a mean loss of 1.1 kg within the first four weeks of a six-week course of chemoradiotherapy [7]). Notably, the impact of low muscle mass for people with lung cancer is clinically meaningful, with a meta-analysis of 15 studies across a range of treatment modalities finding a 3.13 higher risk of mortality in those with low muscle mass [9]). This is in the context of a median survival of less than 2.5 years following curative intent chemo-radiotherapy [10]).

Despite the well-established and serious impact of low muscle mass and muscle loss on survival outcomes in adults with NSCLC, it is unclear how this affects the ability of people with lung cancer to cope with treatment and the impact on their day-to-day functioning. Reviews of nutrition and physical activity interventions for people with lung cancer report some evidence of a benefit on outcomes such as nutritional status, muscle mass, muscle strength and physical function [11, 12]). However, there is a limited understanding of awareness of the role of nutrition and exercise and how it is perceived among people with low muscle mass or muscle loss undergoing treatment for lung cancer. Therefore, the aim of this study was to explore the experiences of patients living with low muscle mass or muscle loss during curative intent treatment for lung cancer. Additionally, we sought to identify receptiveness to and preferences for nutrition and exercise interventions to manage these conditions.

## Materials and methods

This paper reports on the qualitative component, using individual semi-structured interviews, of a larger mixed methods observational study that was designed to identify predictors of muscle loss and the association with treatment outcomes [13]). This project was approved by the Human Research Ethics Committee at Peter MacCallum Cancer Centre (HREC/53147/PMCC-2019) and Deakin University (2019–320). This study is reported according to the Consolidated Criteria for Reporting Qualitative Research (COREQ) checklist [14]). Interviews were conducted between April 2021 and September 2023.

### Participants and recruitment

Participants in the larger study (Predicting muscle loss during lung cancer treatment: PREDICT) were adults with a diagnosis of NSCLC or SCLC treated with curative intent standard dose and fraction chemo-radiotherapy or radiotherapy alone. Participants were recruited by research assistants from lung cancer clinics at three tertiary hospitals across Victoria, Australia between 23^rd^ September 2019 to 30^th^ April 2023. All participants provided written informed consent. These hospitals represent a specialist cancer centre, metropolitan and regional hospital with radiotherapy centres treating patients with lung cancer.

Purposive sampling, aiming to recruit 15 to 20 patients, was used whereby participants who presented with low muscle mass prior to treatment (baseline) or at two to three months after treatment (follow-up), or those who experienced any degree of muscle loss over the duration of the study, were eligible to participate in a phone interview.

Computed tomography (CT) image analysis at the third lumbar vertebrae was used to identify skeletal muscle cross sectional area (CSA, cm^2^) that was normalised for height to determine skeletal muscle index (SMI cm^2^/m^2^), with low muscle mass identified using pre-defined cut-points by Martin et al (< 43 cm^2^/m^2^ in men with a BMI < 24.9 kg/m^2^, < 53 cm^2^/m^2^ in men with a BMI > 25 kg/m^2^, and < 41 cm^2^/m^2^ in women of any BMI) [15]). Routine CT images taken for diagnostic (pre-treatment) and treatment evaluation (post-treatment) purposes were utilised. Loss of muscle mass was considered to have occurred if participants had any reduction in CT-defined skeletal muscle CSA (cm^2^) on the post-treatment CT relative to the pre-treatment CT. Participants who met these criteria were asked by the research team at the final study assessment if they would be interested in participating in an interview. If participants agreed, their name and contact details were provided to the interviewer (NK). The interviewer then reached out to explain the interview process and answer questions, and for those interested, schedule an interview.

### Data collection and analysis

Demographic information (age, sex, living situation) and clinical information (diagnosis, disease stage, treatment) were collected from the medical record. Anthropometric measures (weight, body mass index) and physical function (Short Physical Performance Battery) were measured at baseline. All interviews were conducted over the telephone following a semi-structured interview schedule (Table 1) by NK who holds a PhD and is a female researcher in the field of oncology nutrition and body composition research and is formally trained in qualitative research. The interview guide was reviewed and revised by all authors who consist of clinicians and researchers in oncology nutrition, exercise, body composition and lung cancer. The questions sought to understand how participants coped throughout treatment and recovery, with completion of self-care, as well as their experience of nutrition and exercise support. Prior to commencing the interview, the purpose of the research was reiterated, participants were informed the interview would be recorded and were given the opportunity to ask questions. Verbal confirmation of consent was obtained and recorded at the beginning of the interview. Only the interviewer (at her workplace) and participant (at home) were present during each phone interview as arranged at the time of scheduling the interview. Participants were unknown to the interviewer at the time of the interview. All interviews were audio recorded and transcribed verbatim.

**Table 1:** Semi-structured interview schedule.

1. Can you tell me about your overall experience of your chemo-radiotherapy/radiotherapy treatment?
2. How did you feel you coped with your treatment?
3. Were there any issues with your strength (or stamina) during or after treatment?
a. Prompt: If so, how did this affect you day to day?
b. Prompt: Were you able to do the things you usually do?
4. Were you given any advice about improving/ maintaining your strength or doing exercise while you were having treatment?
a. Prompt: If so, can you describe who provided the advice and the impact it had on you?
5. Were there any issues with your ability to eat and drink during or after treatment?
a. Prompt: If so, how did this affect you day to day?
b. Prompt: Were there any issues with shopping or preparing meals?
6. Were you given any advice about eating while you were having treatment?
a. Prompt: If so, can you describe who provided the advice and the impact it had on you?
7. Can you tell me about your awareness and views on the role of nutrition and exercise in your treatment?
8. At what time (before, during or after treatment) would advice about nutrition and exercise have been most helpful to you?
a. Prompt: When would you be receptive to this advice?
b. Prompt: Does this differ for nutrition compared to exercise?
9. How would you prefer to receive advice and support to improve nutrition and exercise?
a. Prompt: Individually? From multiple clinicians?

Interviews were analysed using thematic analysis techniques [16]). Analysis of transcripts was commenced alongside data collection to enable identification of data saturation. Transcripts of each interview were read by NK who completed initial open coding which involved assigning labels to sections of the transcripts. For rigour, initial codes were reviewed by AU and LE after completion of three interviews and again upon completion of all interviews to establish agreement regarding codes and facilitate grouping similar codes into themes. The process of analysis was iterative and reflective to understand and interpret the patient experience.

## Results

### Participants

Of the 60 participants recruited to the PREDICT study, 48 (80%) were eligible to be interviewed at two to three months following completion of treatment. Of these, 17 were lost to follow up or withdrew, 2 were too unwell, 3 declined to be interviewed, and in 7 cases the post-treatment CT image that determined eligibility could not be obtained within a suitable timeframe, which resulted in 18 participants being interviewed. The majority of participants were male (61%), lived with others (78%), with a mean age of 73 years, predominantly NSCLC stage III disease and treated with chemo- radiotherapy (Table 2). Over half (61%) had overweight or obesity and the majority presented with low muscle mass prior to treatment (55%) and experienced muscle loss over the duration of treatment (61%) (Table 2). The mean duration of interviews was 21 minutes (range 10–35 minutes).

**Table 2:** Participant characteristics at baseline.

	Total (N = 18)
Age (mean ± SD)	72.7 (9.0)
Sex, n (%)	
Female	7 (39)
Male	11 (61)
Living situation, n (%)	
Alone	4 (22)
With others	14 (78)
Site	
Peter MacCallum Cancer Centre	8
Barwon Health	5
Austin Health	5
Diagnosis and stage	
NSCLC	
Ia	1 (5.5)
Ib	1 (5.5)
IIb	4 (22)
IIIa	6 (34)
IIIb	1 (5.5)
IV	1 (5.5)
SCLC	
Limited	4 (22)
Treatment	
Radiotherapy alone	5 (28)
Chemo-radiotherapy	13 (72)
Body mass index (kg/m^2^)	
< 18.5	1 (6)
18.5–24.9	6 (33)
25.0–29.9	9 (50)
≥ 30	2 (11)
Weight, kg (mean ± SD)	75.4 (14.4)
Physical performance^a^ (mean ± SD)	10.0 (0.9)
Low muscle mass, n (%)	
Yes	10 (55)
No	8 (45)
Muscle loss throughout treatment, n (%)	
Yes	11 (61)
No	7 (39)
Low muscle mass and muscle loss throughout treatment, n (%)	
Yes	4 (22)
No	14 (78)

^a^Scores can range from 0 to 12, with higher scores indicating better performance.

Three themes were evident in the data: 1) the effect of cancer and its treatment; 2) engaging in self-management; and 3) impact and influence of extrinsic factors. These themes and their respective categories (Table 3) are presented with supporting quotes from participants using participant identifier numbers [P1 to P18]

**Table 3:** Summary of themes and supporting codes.

Themes	Codes
(1) The effect of cancer and its treatment	Minimal impact of treatment
	Weight loss and management
	Treatment or cancer-induced side effects
	Lack of advice about nutrition and/or exercise
	Impact of treatment side effects on eating
	Return to usual food enjoyment after treatment completion
	Treatment-related impact affecting day-to-day functioning
	Post-treatment impact on health
	Impact of treatment on ability to return to work
(2) Engaging in Self-Management	Adapting food intake or choices to manage symptoms
	Awareness of the role of nutrition
	Maintaining usual activities
	Awareness of muscle health and impact on strength
	Perception of benefit from exercise
	Adjusting type of physical activity to current ability
	Support for self-directed nutrition and physical activity care
(3) Impact and Influence of Extrinsic Factors	Impact of support from health professionals or family
	Need for health professional support and personalised advice
	Trust in credible sources of diet and exercise advice
	Challenges and considerations in optimal timing of advice
	Consistent messaging from health professionals
	Not learning anything new from health professionals
	Conflict between new information and long-held beliefs
	Importance of early provision of information about diet and exercise
	Reliance on family and friends for support
	Advice not responsive to patient situation

### Theme 1 –The effect of cancer and its treatment

Most participants reported experiencing substantial cancer and treatment-related side effects. This manifested in a number of ways, including the desire and ability eat, capacity to be physically active and function as usual within their daily life. The most commonly expressed side effects were nausea, “*well basically*, *it was just an unbelievable*, *previously unexperienced level of nausea that was 24 hours a day*,*”* (P8); oesophagitis, *“there was this excruciating*, *absolutely excruciating pain and I was taking painkillers*, *and I thought…it was just nearly unbearable*,*”* (P17); and extreme fatigue, *“I’ve been progressively getting very*, *very tired”* (P18).

A small number of participants described experiencing minimal side effects during treatment, *“going through the radiation*, *and the chemo*, *I had no problems whatsoever*, *and I made the comment a couple of times I think they’re just pumping placebo into me because I didn’t feel any different to what I did”* (P3). In some cases, the lack of side effects changed in the period after treatment was completed, *“I was a bit disappointed because I felt a lot better while I was doing treatment*, *than I seemed to be after I finished*, *I was expecting to get better but I actually got worse”* (P5). Participants also described a prolonged period of recovery following the end of treatment, *“treatment finished in end of May I think it was I think it finished*. *It [recovery] took a few months after that”* (P11); and in some cases they were surprised by the length of time involved *“I was being a bit too optimistic while I was having treatment about the recovery times on a lot of the different things*. *That I was expecting to finish the treatment and be up and ready to go and back roughly*, *back to normal in a couple of weeks”* (P15).

Participants spoke about the profound effect of pain related to oesophagitis, taste alteration and reduced appetite on their food intake. In some cases, these side effects resulted in a loss of one of life’s pleasures “*well it’s not the end of the world*, *but it seemed like it at the time*. *I want to do the things that I like most in this world*, *eating and drinking so*, *I didn’t sort of take not eating easily*, *but I mean I just had no desire to eat”* (P8).

Some participants reflected on the gradual resolution of side effects after treatment ended and expressed a return to their usual enjoyment in eating *“when I finished that intensive treatment*, *I was really pleased that my taste buds went back to normal”* (P4). For others, a lack of side effect resolution led to concerns regarding the potential for further impact on their wellbeing “*I thought if I’m not eating my energy levels and strength will start to deteriorate*. *But I thought what can I do about it*? *I can’t really force myself to eat too much because it hurts too much”* (P3). Most participants described a focus on weight as they were going through treatment, with health professionals discouraging and concerned about weight loss, *“that’s when we got to the radiotherapy [the nurses] was flagged about my weight loss*. *So*, *they organised*, *I spoke with the nurses there and then they organised me to speak with the dietitian*, *which was helpful”* (P9). Some shared the concern of their health professionals, *“I think I’m now 49 [kilograms]*, *and as I say desperately trying to put some weight back on because all my clothes are falling down”* (P1). For others the focus on weight and the advice to increase food intake to counter weight loss was unwelcome, *“I thought that’s alright for you to say that*, *but you know*, *I can eat a good three meals a day*, *and it’s still too much*. *I’m not going to lose weight*, *I want to lose a little bit of weight”* (P4).

Many participants described an overwhelming fatigue and loss of stamina that significantly affected their ability to go about their usual daily tasks, *“I used to go and pick some grandkids up and take them to school*, *I just didn’t have the energy for that”* (P11). Some participants reported feelings of weakness, *“I felt really weak in the muscle*, *my muscles I thought what’s going on*? *A couple of days it was hard to get off the toilet”* (P4); and loss of strength, *“I lost muscle*, *muscle power and that’s where it was*, *I didn’t have the strength in the muscles*, *like I picked up a mop and it felt very heavy”* (P14). In some participants this led to a greater need for sleep, *“I could sleep from 6pm to 7am*, *so that’s 13 hours”* (P4). In others, it affected their ability to rest, *“I’d feel tired but I wasn’t tired*. *Like I’d go sit down to try have a snooze and couldn’t have one*, *I wouldn’t go to sleep”* (P11). Participants who were still working at the time of their diagnosis noted a reduced capacity for work, *“I’d try to start work and then just ring the team later and just say*, *look I gotta go and then I’d just go to bed”* (P17); or ability to return to work, *“I was panting a bit just before when I came back up again*, *just coming up one flight of stairs*. *So*, *on a building site*, *there’s no way I could cope with that”* (P5). Similarly, participants reflected on the impact of treatment on their ability to be physically active, *“while a lot of the radiation was going on*, *I just literally didn’t have the energy to do the walking*. *It just zapped me of all of all energy*, *I was actually surprised that it actually affected me that much”* (P15).

Despite these significant issues experienced by participants, several reflected on the lack of access to professional nutrition and exercise advice as they went through treatment, *“No*, *nobody has mentioned that at all” (P15)*.

### Theme 2 –Engaging in self-management

In general, participants had an awareness of factors such as nutrition, exercise and muscle health, the role these play throughout treatment and recovery, and they employed self-management strategies to cope with their situation. Regarding nutrition, for some, improving nutritional intake was specifically seen to be of benefit to restoring lost weight and muscle, *“I knew at the start [about importance of nutrition] because of the muscle wastage and the weight loss*, *which are more or less the same thing*, *not quite*, *close enough*, *but yes*, *something was needed”* (P8). In others, nutrition was perceived as important in relation to general health and wellbeing, *“I eat healthy*. *I really do eat healthy*, *not just now because of this*. *I always all my life I eat healthy”* (P12). Some reported managing symptoms though making modifications to usual meal patterns, *“I changed my main meal to the middle of the day because I don’t ever feel like eating at night”* (P1); or usual food choices, *“I’ve tried different*, *different foods*. *I found that the vegetables and so forth I was really hoping to*, *to struggled to eat any of that*. *Soups weren’t bad*, *the thing that seemed to have gone down better than anything were the sweets”* (P16).

The value of maintaining or regaining muscle strength and health to support a return to usual functioning were appreciated by some participants, *“I really want to get back to gym because I think my muscle tone in general is bad*. *My two-year-old granddaughter throws herself at me and I can hardly lift her*, *you know what I mean over my head sort of thing*. *I think geeze I’m gonna drop her*, *she’s too heavy now*. *So*, *I’d like to get some more muscle strength”* (P4). Most participants recognised the benefit of exercise for their recovery and longer-term health, *“it is important to*, *to keep my movement good*. *It’s just very important*. *Exercise is very important for my health”* (P6). Some participants engaged in some form of exercise throughout treatment and reflected that this helped them cope with treatment, *“I was going swimming probably four days a week… I’d be lucky if I got 10 laps in but just being in the water*, *walking up and down was really good*. *But certainly*, *during chemo doing that walking every day I really believe really helped me a lot*…*physically*, *of course I think that really helped”* (P7). Others reflected on a desire to start or re-engage with exercise following the completion of treatment as they begin to feel well again, *“but as soon as I got my energies back up and if I’ve got time before I go away I’ll start up at the*, *the [exercise] studio again*, *and he’ll put me back on strength training exercises again”* (P15). Adjusting the type or duration of exercise compared to usual was a strategy utilised by some participants to maintain some form of exercise within their current ability, “I can’t exercise hard anymore. I mean, the body is not as pliable as what it used to be”(P18).

In the absence of more structured exercise, some participants described a focus on staying physically active throughout treatment by continuing usual household chores, *“well*, *I did stay reasonably active*. *I still had my normal jobs to do around the garden and stuff”* (P13). Others were offered exercise programs within their health service but expressed a preference to continue their own previous activities, *“no*, *no*, *I said no*. *I said I’ll do a bit of exercise because I always used to do swimming before this happened*. *I used to do a lot of swimming”* (P10). The concept of the health service providing resources for people to engage in self-directed nutrition and physical activity management was raised by a small number of participants. For some, reluctance to engage in self-directed management was due to a feeling of being overwhelmed by their situation, *“because when you’re not feeling well*, *and you’re feeling crook as a dog*, *and someone’s telling you about nutrition you don’t give a flying… about it”* (P17); and a preference to refer to written information when it was expressed, “*then when you’re not feeling well*, *you’ve got the pack*, *you can have a look at it at your leisure”* (P17). In others having the option of self-directed care allowed them some control over the elements of care they wished to engage with, “*they offer the advice but that it’s not imposed on you*. *So*, *I think that’s how it is*, *just don’t impose on me that I have to do it"* (P14).

### Theme 3 –Impact and influence of extrinsic factors

This theme represents the numerous extraneous influences participants encountered throughout treatment and the impact of these on their ability to cope with and navigate the treatment process. The role of health professionals, family and friends in providing support was perceived as vital. Participants reflected on the impact health professional support had on their decision making and motivation, *“We had a great instructor [in an exercise group]… and he was wonderful*. *If you couldn’t do it [an exercise] or you felt it was hard to do*, *he’d come over and explain to you how to not feel that way’* (P2). Many participants spoke of the crucial role family and friends held in assisting them through daily tasks and motivating them to undertake self-care, *“… it’s difficult to do exercise*, *you don’t feel like it*, *for me I didn’t feel like doing it*. *So that the family support comes in and they try to help you to go for a walk and things like that*, *that you need to do”* (P9). However, some reflected on a need for a level of support beyond that which family could provide, *“They’re quite a distance from me and yeah*, *so*, *I will say that there were times where I felt I could have done with a lot more support”* (P7). Hearing consistent messages regarding nutrition and exercise from health professionals was viewed favourably in terms of maintaining self-care activities, *“any time I asked anyone what can I do to help myself*, *the advice was keep*, *keep exercising you know and good diet*, *good diet and exercise…”* (P4). Although in some cases, participants described a feeling of not learning anything new from the advice received from health professionals and felt their own knowledge was sufficient, *“Wasn’t all that important*, *we knew what we had to eat”* (P13).

While the support received from health professionals was valued highly, participants concurred that it was important this advice was personalised to their situation, *“it’s going to be case by case because you’re dealing with all sorts of*, *you’re treating different things”* (P8). Furthermore, there was an inherent trust and credibility in information about nutrition and exercise that came from health professionals in the treating team, *“*…*it [advice from health professionals] sort of made it more important and imprinted it better I think”* (P10). For some, the opportunity to see a health professional in person and discuss issues that were specific to their circumstances was preferred to being provided with written information “*You could raise questions with her and talk about different issues that you had*. *You can’t do that when you read something”* (P3).

However, some participants described less positive experiences with their health professionals, where the advice they were receiving was not responsive to their current circumstances and was a source of frustration, *“there’s no question that I needed and I’m still taking dietary supplements*. *No question about that*, *but anything else just makes no sense*, *sitting*, *trying to figure out ways to get food down your throat that will not swallow”* (P8). Similarly, other participants spoke about being given advice about eating during treatment that conflicted with their own understanding and long-held health beliefs about nutrition. In some cases, this related to the message from dietitians and other health professionals to avoid losing weight throughout treatment, *“I kept saying to my husband*, *I wished I’d lost weight because they kept saying make sure you eat*, *we don’t want you to [lose weight]*, *and I said I would love to”* (P10). In other cases, these concerns related to the types of high energy food choices that were recommended by some health professionals, *“that just goes against everything that I have experienced in that eating that kind of food is just going to make you feel really crap and nutritionally why are they encouraging people to eat that kind of food*?*”* (P7).

There was a consistent and strong perception that providing advice about nutrition and exercise as early as possible in the treatment process alongside ongoing support was important. Participants reflected on being more receptive to information early in their treatment prior to the onset of treatment-related side effects, *“The more information you get*, *the earlier you get it*, *the better I think”* (P5). In addition, the value of maintaining support and advice about nutrition and exercise was recognised, *“you sort of forget*, *you get lazy*, *and then you get in your own little*, *you know*, *rut in your life*, *where you do what you do and eat what you eat*, *but probably just a little nudge to help remind you that the body is going to need that bit extra care”* (P10).

While recognising the need for advice on nutrition and exercise, several challenges were raised in relation to the optimal timing and delivery. In many cases this related back to the overwhelming impact of treatment side effects such as fatigue, *“I’ve had no energy*, *during [treatment] I*, *it was 100% in my head*. *No man I was*, *I was very rapidly losing [weight] and I don’t think I could’ve lasted much longer”* (P8); and loss of appetite, *“if you don’t feel like eating*, *you’re not going to eat so someone can tell you about nutrition till the cows come home and if you’re not feeling like it*, *you’re not going to do it”* (P17). In others, the timing of advice was crucial to how it was received, *“Not in a great receptive mood for anything straight after*, *believe me please*. *You’re just not*, *so find whatever*, *find the time in a day that’s before the chemo*, *not after*. *I mean I think that probably is critical if you’re going to want anything listened to”* (P8).

## Discussion

This study explored the experience of people with lung cancer undergoing chemo-radiotherapy or radiotherapy treatment while living with low muscle mass or muscle loss. Although a variety of experiences were described, overall participants reported a substantial burden from cancer and treatment-related side effects. Side effects such as fatigue, nausea and oesophagitis impacted their day-to-day functioning, nutritional intake and ability to participate in exercise or physical activity. Participants were aware of the purpose of nutrition and exercise in supporting physical health to cope with treatment. Participants utilised self-management strategies that were self-initiated or recommended by health professionals to improve or maintain nutritional intake and participation in exercise or physical activity. Although, difficulties in achieving this were expressed due to the high symptom burden. The need for support throughout treatment from health professionals and carers was high. Furthermore, the importance of personalised care that considered other factors that were affecting ability to engage with nutrition and exercise advice was valued. The need to receive information early in the treatment timeline about nutrition and exercise, and how to access these services, was consistently conveyed, although preferences for how this was communicated varied.

The debilitating symptom burden associated with lung cancer and its treatment is well established [3]) and reinforced by participants in our study. What had not previously been explored is the impact of this symptom burden in patients with low muscle mass or muscle loss. Adequate nutritional intake is essential to prevent or attenuate loss of muscle mass [17, 18]), yet participants were describing severe oesophagitis and nausea and frustration at health professional attempts to encourage continued oral intake in this situation. Similarly, resistance exercise is a key strategy to mitigate muscle loss as a consequence of cancer and associated treatments [18, 19]), although in our study symptoms of fatigue reduced capacity to complete usual household tasks as well as participation in exercise. Strategies to support patients with low muscle mass in the adoption of nutrition and exercise interventions that may help them cope better with lung cancer treatment require further investigation.

Despite most participants expressing an understanding that nutrition and exercise were important aspects of care throughout (chemo)radiotherapy, there are clear areas where further clarity could be provided by health professionals to facilitate improved understanding. Across the interviews, there was a significant focus on weight management and avoiding weight loss, reflecting advice received from health professionals due to the known adverse prognostic outcomes from unintentional weight loss in people with lung cancer [20]), and this was not always well-received. An increasing proportion of people (>50%) are presenting with overweight or obesity at the time of a lung cancer diagnosis [7, 12]) and for some the weight loss associated with treatment and side effects may be viewed as a welcome opportunity to reduce weight. This emphasis on weight fails to reflect contemporary understanding of body composition and the prognostic significance of low muscle mass and muscle loss [6]). Furthermore, it has been demonstrated in people with colorectal cancer that significant underlying muscle loss can occur despite weight stability [21]). A shift in the focus of health professional advice to preserving muscle mass and strength, rather than weight, may be more meaningful for people with overweight or obesity, particularly if this equates to improvements in fatigue and physical function [22]). Similarly, some participants reported surprise regarding the types of high energy and high protein foods recommended to prevent weight loss during treatment, which in some cases included discretionary foods. Clinical guidelines for nutrition and cancer recommend an energy intake of between 25 and 30 kcal/kg/day and protein intake of 1.0 to 1.5 g/kg/day during cancer treatment [17]). However, this can be challenging to achieve due to anorexia and other symptoms experienced during treatment [23]) and therefore dietitians may recommend energy dense food and fluid choices which may conflict with patients views of appropriate food choices [24]). The optimal food choices and dietary patterns to preserve muscle mass during cancer treatment is the subject of ongoing research [25, 26]).

Access to nutrition and exercise services for people undergoing cancer treatment varies widely [5, 27]). In our study, participants indicated a preference for information about nutrition and exercise to be provided early in treatment, prior to onset of symptoms, yet some reported a lack of access to these services entirely. Barriers to participating in exercise, but not nutrition therapy, during (chemo)radiotherapy for lung cancer have previously been described and include symptom exacerbation, prioritising other tasks ahead of exercise and demanding treatment schedules [28]). Similar concepts arose in our study, with participants stating the timing of advice was critical and at times they were not receptive to this due to symptom onset or fatigue from a multitude of appointments. The preferred means by which nutrition and exercise services could be accessed varied, with some people favouring written or online guidance they could refer to in their own time and others face-to-face contact with a health professional. In view of the multiple benefits of nutrition and exercise throughout treatment [18, 19]), the challenge for health services is having the flexibility of programs that cater to individual preferences. At a minimum patients should be made fully aware of the benefits that may be gained from nutrition therapy and participation in exercise to make an informed decision about which aspects of care they want to access.

This study has several strengths and limitations. The participants represented diversity in age, living situation, disease stage and body mass index and were recruited from three tertiary cancer treatment centres located in metropolitan and regional areas. To our knowledge the experience of patients with lung cancer living with low muscle mass or muscle loss while undergoing (chemo)radiotherapy has not previously been explored and provides important information that may guide the delivery of care. The timing of the interviews at two to three months post-treatment meant that participants who were approached to be interviewed were those who remained in the study and may reflect participants who were generally coping better with treatment. The three health services where recruitment occurred varied in the resourcing and level of access available for people with lung cancer to nutrition and exercise services and is likely to have resulted in different reflections and experiences. Although our published protocol initially aimed for diversity in participant characteristics, due to recruitment challenges we did not focus on specific participant groups.

A diverse range of experiences and ability to cope with treatment were described by participants. Multiple factors affected the ability to engage with nutrition advice and exercise, while the opportunity to access these services varied. Awareness of the role of nutrition and exercise in their treatment was high among participants, although with a strong focus on preserving weight rather than muscle mass. Several implications for the delivery of nutrition and exercise support to patients with lung cancer and low muscle mass are apparent. A shift in focus from discussions around unintentional weight loss to preservation of muscle mass should be considered and the functional benefits of mitigating muscle loss should be communicated to patients. Strategies to improve the capacity of patients to participate in exercise and nutrition therapy are required. The types of support required were highly individual, highlighting the crucial role of personalised identification of needs and subsequent intervention.
